# Breastfeeding in Saudi Arabia: a review

**DOI:** 10.1186/1746-4358-9-1

**Published:** 2014-01-14

**Authors:** Daifellah A M Al Juaid, Colin W Binns, Roslyn C Giglia

**Affiliations:** 1 College of Applied Medical Sciences, Taif University, PO Box 888, Taif 21974, Saudi Arabia; 2 School of Public Health and Curtin Health Innovation Research Institute, Curtin University, Perth, Western Australia 6845, Australia; 3 School of Public Health, WA Centre for Health Promotion Research, Curtin University, Perth, Western Australia 6845, Australia

**Keywords:** Breastfeeding, Breastfeeding duration, Epidemiology, Infant feeding, Saudi

## Abstract

**Background:**

Breastfeeding is viewed as the optimal method of infant feeding that provides many benefits to both the infant and the mother. The monitoring and reporting of breastfeeding indicators are essential for any country to plan and implement effective promotion programs for sustainable breastfeeding. The aim of this review is to examine the available studies and data on breastfeeding in Saudi Arabia, and determine the potential factors that affect breastfeeding practices and duration in this country.

**Methods:**

The databases of Web of Knowledge, Science Direct and PubMed were searched using the relevant key words. Only studies that reported breastfeeding practices, rates and indicators in Saudi Arabia were included. Standard WHO definitions for breastfeeding categories were used in this review.

**Results:**

Seventeen cross-sectional studies were identified and reviewed and five stated they used standard definitions. The self-administered questionnaire as a measurement tool was the predominant method of data collection. Infants' ages range from less than six months up to five years. Initiation rates were high (mostly above 90%), but a few studies reported low rates of timely initiation (within the first hour). The exclusive breastfeeding rate could not be accurately determined as rates range from 0.8% to 43.9% among studies due to the lack of clear definitions and the nature of study design. The partial (mixed) feeding method was common and the category of 'any breastfeeding' has generally high rates. The mean duration of breastfeeding has showed a progressive decline over time from 13.4 months in 1987 to 8.5 months in 2010. Factors associated with a high prevalence of breastfeeding and longer duration include increased maternal age, low educational levels, rural residence, low income, multiparity and avoiding contraceptives. The most common reason for breastfeeding cessation was insufficient breast milk. Other reasons include sickness, new pregnancy and breastfeeding problems.

**Conclusions:**

Breastfeeding indicators in Saudi Arabia could not be monitored or compared relying on the available data because no longitudinal studies have been conducted in this country. A cohort study design would be the most appropriate procedure to rigorously assess and report valid results on breastfeeding practices and patterns in the Saudi society.

## Background

Breastfeeding is the optimal method of infant feeding bringing short- and long-term benefits for infants, mothers, environment, economy and the entire society [1-3]. The World Health Organization (WHO) and other international health bodies have recommended exclusive breastfeeding for six months after birth [4-6]. It is also recommended that breastfeeding continues for two years or longer together with nutritionally-adequate complementary foods [2,3].

The Eastern Mediterranean Regional Office of WHO (EMRO) has reported high rates (>60%) of early breastfeeding initiation with 60% of mothers continuing to breastfeed to 12 months in the Middle East and North Africa (MENA) countries [7]. Despite these recent reports of high rates, previously low rates of exclusive breastfeeding had been reported from this region where only 40% or less of infants under six months were being exclusively breastfed [7]. Dop and Benbouzid [8] reported that the mean rate of ‘exclusive breastfeeding’ at four months in the Middle East region is 24%, including Lebanon (7%), Yemen (15%), Pakistan (16%), Jordan (32%) and Iran (48%). The Global Data Bank on infant and young child feeding (updated in 2009) contained low ‘exclusive breastfeeding’ rates from the MENA region. These low rates have been observed in countries such as Algeria (10.4% at four months and 6.9% at six months), Sudan (21.4% at four months and 15.6% at six months) and Egypt (30.3% at six months) [9].

The Kingdom of Saudi Arabia (KSA) has a population of 27 million and an area of two million square kilometers [10]. About one quarter (24.8%) of the population live in Riyadh, the capital city of KSA, the Western region (Jeddah, Makkah and Taif) is inhabited by another 24%; the Eastern Province by 13.3% and the remaining spread over the rest of the country [10]. Saudi Arabia is a high-income country and the government spends 6.5% of the national income on health, with USD 345 of health expenditure per capita in 2010 [11]. Life expectancy is 69 years for males and 75 years for females [12].

There is insufficient data available on breastfeeding in Saudi Arabia to monitor progress and develop promotion programs. The World Health Organization does not report any breastfeeding data in the country profile because there are no national data on breastfeeding [11,13]. A previous review on breastfeeding in Saudi Arabia (2003) documented the deficiencies in statistics with incomplete and inconsistent official data and the lack of uniformity in research on breastfeeding [14]. Because of the public health and clinical significance of breastfeeding, after a decade it is appropriate to further review the data that are available.

The objective of this review is to provide a summary of breastfeeding patterns, practices, rates and duration in Saudi Arabia from the published literature. The review will also include an outline of factors associated with breastfeeding practices as well as reasons for the discontinuation of breastfeeding in the KSA.

## Methods

**Figure 1:**
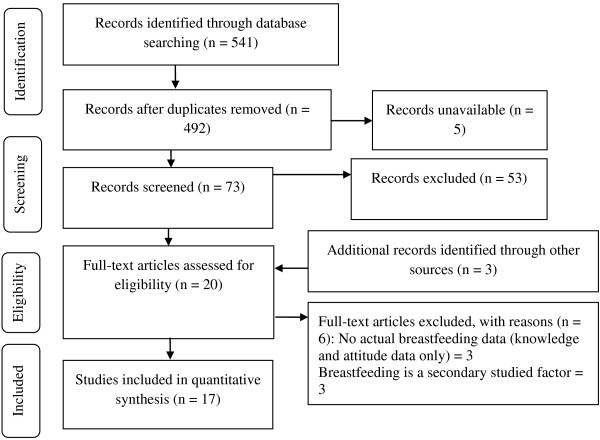
Process of selecting the studies included in this review.

All reported studies on breastfeeding in Saudi Arabia that have been published in the English language until present were sourced. A literature search was undertaken using the Web of Knowledge, Science Direct and PubMed databases. These databases were searched using the key words: Saudi, breastfeeding, breast-feeding, breastfeeding and infant feeding. Where the study appeared to be relevant by including information on the breastfeeding practices in KSA and to document rates and duration, the full text was obtained. The reference lists from obtained full texts were used as additional sources to identify additional relevant studies. Papers concerning knowledge, beliefs and attitudes of women towards breastfeeding were excluded if they did not report the actual practices and rates. Also, studies that investigated related issues such as birth interval, unilateral breastfeeding and caesarean section where breastfeeding was only a secondary associated factor were excluded. The PRISMA diagram in Figure 1 shows the process of selecting studies that were included in this review [15]. The following definitions, which are adopted by the WHO, are used in this review [16,17]. Where a paper claimed to be reporting exclusive breastfeeding, but did not conform to the WHO definition of exclusive breastfeeding it was given the most appropriate classification.

‘*Exclusive breastfeeding*’: allows the infant to receive breast milk only, with no other liquids or foods, not even water, except drops of syrups, vitamins, minerals or medicines.

‘*Predominant breastfeeding*’: allows the infant to receive mixed feeding of breast milk and other liquids, solid or semi-solid foods.

‘*Any breastfeeding*’: allows the infant to receive breast milk (including milk expressed or from a wet nurse) with any foods or liquid, including non-human milk or formula.

When a study used the mere term “breastfeeding” without classifying infants to the above-mentioned categories, it was dealt with under ‘any breastfeeding’ definition in this review.

**Figure 2:**
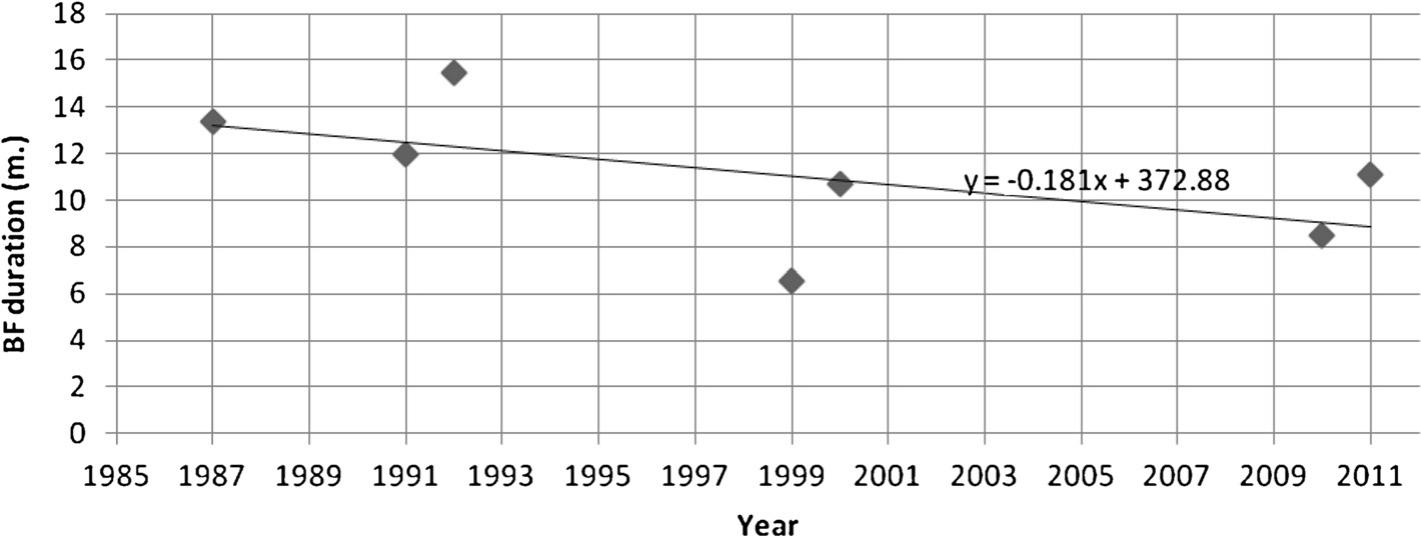
**The decline in breastfeeding duration in Saudi Arabia since 1985, derived from identified studies.**

The values of the mean breastfeeding duration that were reported in the studies were entered to Microsoft Excel® version 2010 to generate a graph and linear regression equation (Figure 2) illustrating the trend of breastfeeding duration over years. There were only seven studies that reported mean breastfeeding duration [18-24]. The seven means of breastfeeding duration were entered to Microsoft Excel® as values of Y-axis, and the years of the seven studies were entered as values of X-axis. Using the features in the Microsoft Excel®, a scatter plot was generated and a linear model was fitted to demonstrate the trend in the breastfeeding duration over time (Figure 2).

All studies included in this review have been assessed against the National Health and Medical Research Council (NHMRC) levels of evidence and ranked according to their designs [25]. NHMRC levels of evidence range from I, the strongest to IV, which is the weakest [25].

## Results

### Breastfeeding definitions and measurement

The search revealed 17 studies, which met the inclusion criteria, and Table 1 provides information on the definitions of breastfeeding categories they used and levels of evidence they provide. Only five of studies stated that they used the WHO definitions for breastfeeding categories in their methodology sections [21,22,26-28]. Studies that have not used standard definitions might misclassify infants into incorrect breastfeeding categories, thus the initiation rates, breastfeeding rates (particularly exclusive breastfeeding rates) and breastfeeding duration may be overestimated [17].

**Table 1:** Definitions and categorisation of infants as reported in the identified studies

**Study**	**Definitions – infant feeding categories**	**NHMRC level **[25]
Serenius, 1988 [29]	No definitions provided	IV
Al-Othaimeen, 1987 [35]	No definitions provided	IV
Al-Mazrou, 1994 [18], p. 267	“The term weaning was used to denote the event of stopping of breastfeeding. Supplementation meant the addition of other milks or semi-solids to a breastfed baby”.	IV
Madani, 1994 [30]	No definitions provided	IV
Al-Shehri, 1995 [20], p. 41	“Breastfeeding only refers to those infants who were breastfed exclusively without any reconstituted powdered milk or any other infant formula. Bottlefed refers to the infants and children who were given reconstituted powdered milk or other infant formula”.	IV
Kordy, 1992 [24]	No definitions provided	IV
Shawky, 2003 [31], p. 92	“If the mother lactated only or breast fed together with bottle or solid foods, breast feeding was considered to be still continuing”.	IV
Al-Ayed, 1998 [32], p. 114	“Infants were grouped into the following broad categories for analysis: exclusively breastfed (breast + semisolids); exclusively bottle fed (bottle + semisolids), and infants on mixed feeding (breast + bottle + semisolids)”.	IV
Al-Jassir, 2004 [19]	No definitions provided	IV
Khattab, 2000 [23]	No definitions provided	IV
Fida, 2003 [33]	No definitions provided	IV
Al-Jassir, 2006 [34]	No definitions provided	IV
El Mouzan, 2009 [27], p. 21	“According to the WHO definition, exclusive breastfeeding means no other food or fluids (including plain water and juices). Infant milk formulas are considered complementary food”.	IV
Al-Hreashy, 2008 [26], p. 428	“The WHO definitions for breastfeeding were adopted for classification of infant feeding patterns”.	IV
El-Gilany, 2011 [28], p. 209	“According to the World Health Organization definition, exclusive breastfeeding means no other food or fluids (including plain water and juices), and the infant consumes human milk with no supplements of any type except for vitamins, minerals, and medications”.	IV
Amin, 2011 [21], p. 60	“Breastfeeding definitions used in this study were according to the infant feeding recommendations of the 2001 WHO Expert Consultation and the 55th World Health Assembly”.	IV
Eldeek, 2012 [22], p. 157	“World Health Organization (WHO) definitions were used for classification of infants’ nutrition patterns”.	IV

Samples were often recruited during visits to primary health care centres (PHCCs) for vaccination or during home visits. Almost all studies used self-administered questionnaires to report and measure breastfeeding practices from participating mothers. These questionnaires collected data on socio-demographic variables, breastfeeding patterns and practices, infant attributes, and reasons for breastfeeding cessation. Some data, including delivery mode and birth weight were collected from health records of mothers and infants at hospitals and PHCCs. All of these studies were cross-sectional, and data were usually collected relying on mothers’ memory, with the possibility of recall bias. The age range of infants included in the studies (and hence length of recall) varied across a wide range of ages. Four studies [18-20,29] asked mothers about feeding their infants in the last five years, four studies [21,23,24,27] included infants less than 2.5 years, and five studies investigated feeding practices in infants aged less than 12 months [30-34]. Three studies [22,26,28] included infants 6 months or younger, while one study included infants and children 0–9 years old [35]. No prospective cohort studies have been published.

### Breastfeeding rates

Breastfeeding initiation, ‘exclusive’ and ‘any breastfeeding’ rates at selected age ranges and the mean duration as reported in these studies along with times, sites and sample sizes used in the studies are summarised in Table 2. Initiation rates were above 90% in almost all of the identified studies. One study found a considerable difference between urban and rural communities in initiation rates (90% for rural versus 76% for urban groups) [29]. In time to initiation of breastfeeding, El-Gilany et al. [28] reported that only 11.4% of mothers started breastfeeding within the first hour after delivery while Amin et al. [21] found that 77.8% of studied mothers had initiated breastfeeding within 24 hours postpartum.

**Table 2:** Results of studies that have investigated breastfeeding in Saudi Arabia

	**Study year & location**	**Study design**	**Sample size (n)**	**Initiation rate (%)**		**Child age (m**^**†**^**)**	Exclusive BF^* ^**(%)**	**Child age (m**^**†**^**)**	Any BF^* ^**(%)**		BF^*^duration (m^**†**^**)**
									R^**^	U^**^	
1	1979-81, Central & South-West KSA (Serenius 1988 [29])	Cross-sectional	2196	90	R^**^	-		1	90	76	Median=
				76	U^**^			3	90	42	17.8 R^**^
								6	85	22	2.1 U^**^
2	1987, National (Al-Othaimeen 1987 [35])	Cross-sectional	767	89.9		<6	4.4		29		-
						6-	5.6		15		
						12-	9.1		11		
3	1987, National (Al-Mazrou 1994 [18])	Cross-sectional	6086	90.1		1	55		95		Mean = 13.4
						3	36		92		14.4 R^**^
						6	33		88		12.3 U^**^
4	1990, Taif (West) (Madani 1994 [30])	Cross-sectional	1019	98		≤12	43.9		56.1		-
5	1991, National (Al-Shehri 1995 [20])	Cross-sectional	6308	93		<5	53		87		Mean=
						6-12	38		67		13 R^**^
						12+	18		24		11 U^**^
6	1992, Makkah (West) (Kordy 1992 [24])	Cross-sectional	476	97.1		<36	8.9		42.4		Mean=
											14.61 ± 3.53
7	1997, Jeddah (West) (Shawky 2003 [31])	Cross-sectional	400	94		-		6	54		Median = 6
8	1995, Riyadh (Central) (Al-Ayed 1998 [32])	Cross-sectional	347	-		6	22.1		51.6		-
9	1999, Riyadh (Central) (Al-Jassir 2004 [19])	Cross-sectional	21507	98.9		4-6	0.8	6	34.3		Mean=
											6.57 ± 5.71
10	2000, Abha (South-West) (Khattab 2000 [23])	Cross-sectional	100	-		<24	37		47		Mean=
											10.7 ± 6.9
11	2001-02, Jeddah (West) (Fida 2003 [33])	Cross-sectional	128	95		-		≤12	82.8		-
12	2002-03, National (Al-Jassir 2006 [34])	Cross-sectional	4872	91.9		3	23.9	4-6	50		-
13	2004-05, National (El Mouzan 2009 [27])	Cross-sectional	5339	91.6		Birth	70.8		88.8		-
						1	39		49		
						4	16.4		20.5		
						6	8		10.2		
14	2005, Riyadh (Central) (Al-Hreashy 2008 [26])	Cross-sectional	578	95		6	1.7		94.3		-
15	2009, AlHassa (Eastern) (El-Gilany 2011 [28])	Cross-sectional	1904	91.9		6	24.4	-			-
16	2010, AlHassa (Eastern) (Amin 2011 [21])	Cross-sectional	641	91		Birth	66.5		77.8		Mean=
						2	32.9		76		8.5 ± 7.4
						4	19.2		67		Median=
						6	12.2		61		6
17	2011, Jeddah (West) (Eldeek 2012 [22])	Cross-sectional	600			≤6	25		58		Mean=
											11.1 ± 6.64

*BF = breastfeeding, *m*^†^ = months, R^**^ = rural, U^**^ = urban.

Because the vast majority of identified studies were of cross-sectional design and did not provide a standard definition for ‘exclusive breastfeeding’, the rate of ‘exclusive breastfeeding’ in Saudi Arabia could not be determined. However, those studies which used the WHO definition reported that the ‘exclusive breastfeeding’ rate at six months of age ranged from 1.7% [26] to 24.4% [28]. Other studies found low rates of ‘exclusive breastfeeding’ at six months after birth: 0.8% [19]; 8.9% [24] and 5.6% [35]. On the other hand, two national surveys recorded relatively high rates of ‘exclusive breastfeeding’ at six months of age of 33% and 38%, respectively [18,20]. Also, two other studies found that this rate was 37% in children under 24 months [30], and 43.9% in infants less than 12 months of age [23]. Therefore, the prevalence of ‘exclusive breastfeeding’ in the KSA is inconsistently reported and comparisons with the WHO and other international organisations’ recommendations cannot be made because of the weakness of study design used in these investigations.

Mixed (partial) feeding (breastfeeding combined with bottle feeding) has been very common among the Saudi mothers compared to other feeding methods as reported in many of the studies [14,34]. For instance, Al-Othaimeen et al. [35] documented that 57.9% of infants and children under 18 months had received breastfeeding along with artificial infant formula by bottle and glass while only 21.5% and 20.6% of these subjects were exclusively breastfed or bottle-fed, respectively. The ‘mixed breastfeeding’ rates reported by other studies were 88.6% at birth [27], 49.8% at six months after birth [21] and 56% of all infants and children less than two years old [18].

However, Al-Shehri et al. [20] found that 44% of studied infants and children (under five years, n = 4773) were bottle-fed only and 28% were breastfed only whereas only 16% of them were on breast and bottle together and 12% were weaned.

In this review, despite the deficiencies in study design, most of the studies documented high rates of ‘any breastfeeding’ (Table 2). In a recent national survey, El Mouzan et al. [27] reported a rate of only 10.2% for ‘any breastfeeding’ and 8% for ‘exclusive breastfeeding’ among infants aged six months. In contrast, another recent study conducted in the central region (Riyadh) stated that the corresponding rates were 94.3% and 1.7%, respectively [26].

This significant variation in results is an illustration of the inconsistent findings resulting from the absence of appropriate study design, including length of recall, sample size and selection.

Breastfeeding duration appears to have declined over the past 25 years. Although the graph of the ‘mean breastfeeding duration’ based on the seven studies that calculated it [18-24] does not show a perfect linear regression, there is a declining trend in the ‘mean breastfeeding duration’ over time (Figure 2). While the ‘mean breastfeeding duration’ was as high as 13.4 months in 1987, it has dropped to only 6.8 months in 1999 and 8.5 months in 2010 (Table 2 and Figure 2). These findings, however, can be considered only indicative because of the variation in the study samples and locations between included studies.

### Factors associated with breastfeeding practices

#### Maternal age

Fifteen studies examined the effects of maternal age on breastfeeding practices and duration. Just over half of these studies (eight papers) concluded that the prevalence of breastfeeding was higher with longer duration among older mothers compared to their younger counterparts [18,20,21,23,24,29,34,35]. Results from a recent study revealed that increased maternal age was significantly associated with early initiation of breastfeeding (within 24 hours of delivery) (odds ratio [OR] = 2.24, 95% confidence interval [CI] = 1.37-3.68, *p*-value = 0.016); with longer duration (*p*-value = 0.001) and with exclusivity of breastfeeding (OR = 1.14, 95% CI = 1.03-1.23, *p*-value = 0.034) [21]. In a national survey, Al-Jassir et al. [34] concluded that younger mothers tended to introduce solid foods within the first two months, earlier than older mothers (*p* ≤ 0.05). In contrast, six studies found that the effects of maternal age on breastfeeding were not statistically significant [26,28,30-33]. One study found that the mean age of mothers who practiced ‘exclusive breastfeeding’ was 23.4 ± 4.46 years; which was younger than the mean age of those who adopted artificial feeding (29.71 ± 7.89 years) [22].

#### Mother’s education and employment

Education and consequent employment were not common among Saudi women until recently, as old studies reported that the vast majority of subjects were illiterate and non-workers (housewives). For example, Madani et al. [30] found that the proportions of mothers in their sample who were illiterate and not working were 80% and 96.2%, respectively. This trend has changed over time and levels of education and employment have increased due to rapid advancements in economy, education and other social aspects of life in the KSA. In a recent study (2012), it was found that 67% of studied mothers were working and about 91% have had at least intermediate schooling [22].

Four studies concluded that working mothers breastfed less frequently and had shorter duration than non-workers, and that these differences were statistically significant [21,26,28,32]. A further five studies found that working status had no significant effect on breastfeeding practices and duration [23,24,30,31,33], and one study reported a higher ‘exclusive breastfeeding’ rate among working mothers compared to non-working mothers (*p* = 0.005) [22].

Generally, higher levels of education were associated with less breastfeeding, particularly with ‘exclusive breastfeeding’ and a shorter duration of ‘any breastfeeding’ [18-21,24,26,28,30,34,35]. Results from three other studies reported that education has no significant effect on breastfeeding status and duration [23,32,33]. However, sample sizes in these three studies were very small (see Table 2), and thus, their results might be underpowered.

#### Family income and type of residence

Five studies investigated the disparities in breastfeeding practices between rural and urban communities [18,20,21,28,29]. All agreed that breastfeeding was more prevalent among rural mothers with longer duration and later introduction of supplements than urban mothers. These differences in favour of rural areas were statistically significant in three of the five studies. It is worth noting that the majority of rural women in the KSA tend to be illiterate or have a lower level of education compared to their urban counterparts [21,24]. This could support the hypothesis that there might be an association between the level of maternal education and belonging to a rural community, resulting in better breastfeeding practices. Family income was examined by four studies: two of them found no significant association between income and breastfeeding [28,33]. The other two highlighted a decline in breastfeeding with high income [21,32].

#### Other factors

Other factors associated with breastfeeding that have been studied in the KSA include parity, oral contraceptive use and caesarean section. Parity was examined by eight studies. Four found that multi-parity was correlated with a longer duration and higher prevalence of breastfeeding [21,23,26,32]. For example, Al-Hreashy et al. [26] concluded that primiparous mothers have a shorter duration and more likely to introduce artificial formula in the first six months of life compared to mothers of two to four children or those who have more than five children (OR = 0.36, 95% CI = 0.21-0.64 and OR = 0.18, 95% CI = 0.09-0.35; respectively, with primiparous mothers being the reference group). The other three studies did not find any significant association between parity and breastfeeding practices and duration [28,31,33]. Furthermore, in a study conducted in a rural area in Western Saudi Arabia, Kordy et al. [24] stated that higher birth order was associated with shorter duration of breastfeeding (*p* < 0.001).

The use of oral contraception was found to have a negative effect on breastfeeding as reported in two papers by Al-Hreashy et al. [26] (OR = 4.6, 95% CI = 2.8-7.3) and Shawky et al. [31] (OR = 1.5, 95% CI = 1.1-2.2, *p*-value = 0.031). Shawky et al. [31] stated that caesarean section as a method of delivery was associated with shorter a duration (OR = 1.9, 95% CI = 1.3-2.8, *p*-value = 0.001), however, Al-Hreashy et al. [26] found no association (OR = 0.81, 95% CI = 0.45-1.43).

### Reasons for not breastfeeding

Table 3 lists the reasons given by mothers for not breastfeeding at all or breastfeeding cessation after its establishment. The most common reason was ‘insufficient breast milk’ and this reason was reported by about a half of the sample in some studies [19,32,34]. The predominance of such a reason was explained by less breast stimulation, and thus, less secretion of milk due to reduced suckling of the breast when introducing bottle feeding [24,32]. Although physiological deficiency does occur, it is probably not the main cause of perceived milk insufficiency [27,33]. Also, mothers may simply have believed that breast milk alone is not sufficient for their babies’ health [26]. Other reasons include sickness of the mother or child, becoming pregnant, breastfeeding problems, and others. These reasons were given less frequently, but varied considerably in the proportion of mothers across the reviewed papers (Table 3).

**Table 3:** Reasons of not breastfeeding or stopping breastfeeding as reported by reviewed studies

**Study**	**Insufficient milk (%)**	**Sickness of mother or child (%)**	**New pregnancy (%)**	**Breastfeeding problems (%)**	**Others (%)**
1987, National (Al-Othaimeen 1987 [35])	22.97	7.4	67.7	-	1.95
1987, National (Al-Mazrou 1994 [18])	43	11	4	11	21
1991, National (Al-Shehri 1995 [20])	45	9.5	3.5	5	17.5
1992, Makkah (West) (Kordy 1992 [24])	30.9	8.4	27.3	1.25	-
1995, Riyadh (Central) (Al-Ayed 1998 [32])	52.6	-	-	-	27.5
1999, Riyadh (Central) (Al-Jassir 2004 [19])	66.1	4.1	5.1	-	20.6
2001-02, Jeddah (West) (Fida 2003 [33])	50	8.2	1.8	1.8	10.9
2002-03, National (Al-Jassir 2006 [34])	48.3	11.5	13.2	-	8.8
2004-05, National (El Mouzan 2009 [27])	45.5	30.4	-	11.9	12.2
2005, Riyadh (Central) (Al-Hreashy 2008 [26])	49.6	11.2	-	11.6	6.6
2011, Jeddah (West) (Eldeek 2012 [22])	32	19	-	-	3.3

### Other factors not included in the reviewed studies

There were several important risk factors that were not included in the reviewed studies. Maternal obesity is a risk factor for not breastfeeding and for a shorter duration of breastfeeding [36,37]. Nevertheless, previous studies in Saudi Arabia have not addressed the association between breastfeeding practices and other important factors such as maternal obesity (51% among Saudi females in 2007) and Type 2 diabetes (21.4% among females in 2000) [38,39]. Also, factors that are believed to affect breastfeeding such as the type of formulas used, their commercial advertisements, and parental attitudes have not been documented.

### Limitations

This review has several limitations in the data presented on breastfeeding in the KSA. These are mainly related to the paucity of the studies, the small sample sizes and the lack of standard definitions. All studies included in the review are of cross-sectional design and there is a need for more appropriate methods to study breastfeeding, such as cohort designs. Most studies reviewed did not include multivariate analysis and for those that did there was no consistency in the covariates used for adjustment. Furthermore, most studies included lack standard definitions of breastfeeding, and this demonstrates the need for investigations that are based on valid classification criteria.

## Conclusion

Research on breastfeeding in Saudi Arabia to date has been based on cross-sectional study designs and there is a need for cohort studies to more accurately measure breastfeeding and risk factors. The poor study designs and sample selection are reflected in the disparities in reported breastfeeding rates, and particularly in reported ‘exclusive breastfeeding’ rates. The duration of any breastfeeding showed a decline over time, within the limitations of the samples used. Older, less educated and multiparous mothers who lived in rural communities and belonged to the low socio-economic class were more likely to breastfeed and have prolonged duration compared to other groups. The most common cause of breastfeeding cessation and introduction of alternative feedings was insufficient breast milk; a reason that may be more perceived than real.

It is recommended that breastfeeding patterns and practices in the KSA be re-assessed using a more appropriate research design like cohort studies which can analyse follow up data and present more accurate and valid results. This is necessary to inform the breastfeeding promotion programs in this country. It is hoped that this review will serve as baseline information for any upcoming longitudinal studies on breastfeeding in Saudi Arabia or a part of it.

## Competing interests

The authors declare that they have no competing interests.

## Author’s contributions

CB searched the literature, wrote and revised the manuscript. DA searched the literature and wrote the manuscript. RG revised the manuscript. All authors read and approved the final manuscript.
